# Androgen Receptor Signaling Inhibitors in Non‐Metastatic Castration‐Resistant Prostate Cancer in Japan: The ARASHI Study

**DOI:** 10.1111/iju.70235

**Published:** 2025-10-03

**Authors:** Kazuhiro Suzuki, Nasreen Khan, Tomoyuki Taguchi, Kana Hattori, Guifang Chen, Mercedeh Ghadessi, Niculae Constantinovici, Vanessa Quintero, Hiroyoshi Suzuki

**Affiliations:** ^1^ Department of Urology Gunma University Graduate School of Medicine Gunma Japan; ^2^ Bayer HealthCare Pharmaceuticals, Inc. Whippany New Jersey USA; ^3^ Bayer Yakuhin Ltd Osaka Japan; ^4^ Bayer Consumer Care AG Basel Switzerland; ^5^ Department of Urology Toho University Sakura Medical Center Chiba Japan

**Keywords:** advanced prostate cancer, androgen receptor signaling inhibitors, Japanese, real‐world clinical practice

## Abstract

**Objectives:**

To describe clinical use and outcomes of androgen receptor signaling inhibitors (ARSIs) darolutamide, enzalutamide, and apalutamide in patients with non‐metastatic castration‐resistant prostate cancer (nmCRPC) in Japan.

**Methods:**

This retrospective, observational study examined health claims data from acute care hospitals (Medical Data Vision Co. Ltd. database) in Japan, for patients with nmCRPC initiating an ARSI between February 2020 and April 2023. Key outcomes were time to initial ARSI discontinuation and time to progression to metastatic castration‐resistant prostate cancer (mCRPC).

**Results:**

Of 2746 eligible patients, 418 (15%) received darolutamide, 1898 (69%) enzalutamide, and 430 (16%) apalutamide. Median follow‐up was 18.1 months for darolutamide, 20.9 months for enzalutamide, and 24.6 months for apalutamide. The proportion of patients initiating treatment at label dose was 78% (darolutamide), 58% (enzalutamide), and 62% (apalutamide). Median times (95% CI) to discontinuation (unadjusted Kaplan–Meier [KM]) were 16.1 months (13.3–21.0) for darolutamide, 12.3 months (11.3–13.3) for enzalutamide, and 7.1 months (5.3–9.7) for apalutamide. The KM‐estimated proportions of patients progressing to mCRPC by 24 months (95% CI) were 27% (23–33) for darolutamide, 37% (34–39) for enzalutamide, and 40% (35–45) for apalutamide. The results were consistent across inverse probability of treatment weighting sensitivity analyses.

**Conclusions:**

We observed that a higher proportion of patients receiving darolutamide were likely to stay on treatment longer than patients receiving enzalutamide or apalutamide, and progression to mCRPC appeared to be delayed in these patients.

**Abbreviations**
ADT
: androgen deprivation therapy
AE
: adverse event
ARSI
: androgen receptor signaling inhibitor
CI
: confidence interval
CRPC
: castration‐resistant prostate cancer
HR
: hazard ratio
IPTW
: inverse probability of treatment weighting
IQR
: interquartile range
KM
: Kaplan–Meier
mCRPC
: metastatic castration‐resistant prostate cancer
MDV
: Medical Data Vision
MFS
: metastasis‐free survival
nmCRPC
: non‐metastatic castration‐resistant prostate cancer
OS
: overall survival
SD
: standard deviation
SMD
: standardized mean difference

## 1 Introduction

In 2023, prostate cancer was the most common cancer in Japanese men [1]. In 2023, there were an estimated 98 600 new cases of prostate cancer and 14 000 deaths from prostate cancer in Japan [1]. Studies suggest that a substantial proportion (33%) of Japanese patients with non‐metastatic castration‐resistant prostate cancer (nmCRPC) will, within 2 years of diagnosis, develop metastatic castration‐resistant prostate cancer (mCRPC) [2, 3], which is associated with high disease burden, impaired quality of life, and poor survival [4, 5, 6].

The goals of patients with nmCRPC are to delay progression and prolong survival. However, as most patients are asymptomatic, it is essential to consider treatments with a tolerable adverse event (AE) profile [7, 8]. In Japan, three androgen receptor signaling inhibitors (ARSIs) are recommended for the treatment of nmCRPC: darolutamide (approved 2020), enzalutamide (approved 2014), and apalutamide (approved 2019) [9, 10]. Darolutamide, a structurally distinct and potent ARSI, plus androgen deprivation therapy (ADT) significantly improved metastasis‐free survival (MFS) (median MFS: 40.4 months vs. 18.4 months, hazard ratio [HR] 0.41; *p* < 0.001) and overall survival (OS) (HR 0.69; *p* = 0.003) compared with placebo plus ADT [11, 12]. Similarly, enzalutamide plus ADT improved MFS and OS (median MFS: 36.6 months vs. 14.7 months, HR 0.29; *p* < 0.001; and OS: HR 0.73; *p =* 0.001) [13, 14] and apalutamide plus ADT improved MFS (median MFS: 40.5 months vs. 16.2 months, HR 0.28; *p* < 0.001) and OS (HR 0.78; *p* = 0.016) in patients with nmCRPC compared with placebo plus ADT [15, 16]. Rates of discontinuation due to AEs were 9% for darolutamide, 10% for enzalutamide, and 11% for apalutamide versus 9%, 6%, and 7%, respectively, for placebo [12, 14, 15].

There are no randomized clinical trials that compare darolutamide, enzalutamide, and apalutamide head‐to‐head. The DEAR and DEAR‐Ext studies, using US‐based real‐world data from a large network of community urologists, suggested that patients treated with darolutamide had a significantly longer treatment duration and delayed progression to mCRPC compared with those treated with enzalutamide or apalutamide [17, 18]. In DEAR‐Ext (*N* = 1375), the risk of discontinuation was reduced by 27% for darolutamide versus enzalutamide (HR 0.73, 95% confidence interval [CI] 0.61–0.88) and by 31% versus apalutamide (HR 0.69, 95% CI 0.54–0.89) [18]. HRs adjusted for risk factors for mCRPC progression showed a significant 37% reduction in risk for darolutamide versus enzalutamide (HR 0.63, 95% CI 0.50–0.80) and 28% reduction versus apalutamide (HR 0.72, 95% CI 0.53–0.98). MFS was also longer for darolutamide, with the risk of mCRPC progression or death being reduced by 35% versus enzalutamide (HR 0.65, 95% CI 0.53–0.79) and by 28% higher versus apalutamide (HR 0.72, 95% CI 0.55–0.93) [18]. However, no real‐world study comparing ARSIs has been reported in a Japanese population.

The objectives of this study (ARASHI) were to therefore understand the variation in prescribing practices for ARSIs across Japan and to describe the outcomes of these treatment decisions.

## 2 Materials and Methods

### 2.1 Study Design

ARASHI was a retrospective observational study that utilized the Medical Data Vision (MDV) Co. Ltd., database. MDV is an administrative health claims database covering approximately 480 acute care hospitals in Japan. Established in 2003, the MDV database contains standardized healthcare insurance claims data provided by hospitals using the Japanese Diagnosis and Procedure Combination fixed‐payment reimbursement system. Relevant to the nmCRPC population, the database contains a large number of elderly patients and includes health record data from all insurance types (e.g., social, national, and elderly insurance). These data include detailed claims on inpatient and outpatient encounters, such as prescription drugs, medical practice data, and diagnoses. The MDV database has been used previously to assess patients with prostate cancer in Japan [19, 20, 21].

Patients were classified into three cohorts according to their first prescribed ARSI (darolutamide, enzalutamide, or apalutamide) for nmCRPC. The index date was defined as the treatment initiation (claim) date of the first ARSI. This was a retrospective analysis using anonymized data, and therefore informed consent was not required.

### 2.2 Patients

Men aged ≥ 18 years diagnosed with prostate cancer who initiated treatment with darolutamide, enzalutamide, or apalutamide between February 2020 and April 2023 were included in the analysis (Figure S1). Patients were required to have evidence of CRPC (disease code “8848040” or medical/surgical castration) either prior to or within 90 days of initiating the index ARSI.

Patients were followed for a minimum of 6 months after the index date, measured as continuous activity in the database after the index date and up to last data availability, end of study period, or death, whichever occurred first. In the case of death, the follow‐up could be less than 6 months.

Patients were excluded if they had multiple ARSIs recorded at the index date; evidence of ARSI or abiraterone acetate prescription prior to the index date; another cancer diagnosis during the baseline period; and evidence of metastasis any time before or 30 days after the index date (extended to 90 days in sensitivity analysis with consistent results). Metastasis was defined using a validated composite approach. Patients were considered to have metastatic disease at the earliest date when they met any of the following criteria: ICD‐10 codes for metastatic disease (C77.x, C78.x, C79.x, C80.0), progressive prostate cancer (8848066), prostate cancer recurrence (8848074), or mCRPC (8851347); a record of recurrence or advanced staging (M1 or N2+); evidence of therapies indicated for metastatic prostate cancer (abiraterone, docetaxel, cabazitaxel, olaparib, radium‐223, pembrolizumab); or a record of radiation therapy.

### 2.3 Outcomes

The primary endpoint was time to ARSI treatment discontinuation, defined as the time from the index date to the date when the initial ARSI treatment was discontinued. A discontinuation event was defined as the earliest of any of the following events: treatment stop for at least 60 days, switching to another ARSI, or death. A treatment interruption of less than 60 days was not considered a discontinuation event. A sensitivity analysis was performed in which death was treated as a censored event. Another endpoint was time to progression to mCRPC, calculated from index date until the first evidence of mCRPC (as defined above).

A composite endpoint of time to discontinuation or progression was determined, and was defined as the earliest of any of the following events: treatment stop for at least 60 days; switching to another ARSI; death; or progression to metastatic prostate cancer. The proportion of patients initiating the prescribed daily dose of each ARSI was also assessed. The labeled daily doses are 1200 mg for darolutamide (600 mg twice daily), 160 mg for enzalutamide (once daily), and 240 mg for apalutamide (once daily).

### 2.4 Statistical Analyses

All eligible patients identified in the database during the study period were included in the analysis. Continuous variables were described using means, standard deviations, medians, and interquartile ranges. Categorical variables were described using frequency counts and percentages. To assess the balance of baseline characteristics among treatment cohorts, standardized mean differences were reported. Time‐to‐event outcomes were evaluated using unadjusted Kaplan–Meier (KM) estimates for each cohort separately. The proportion of patients with these events, along with the corresponding 95% CIs, was estimated from the KM curves at fixed time intervals (6, 12, and 24 months). Inverse probability of treatment weighting (IPTW) was used to adjust for baseline confounding factors (including age, months from diagnosis of CRPC to index date, cancer hospital designation, and hospital size) among cohorts. A state frequency plot was generated to assess the frequency of patients receiving a specific dose at each time point during the study period, to provide insight into the use of different doses over time. Sensitivity analyses were also performed to assess the robustness of our results.

## 3 Results

### 3.1 Patients and Baseline Characteristics

A total of 2746 patients were eligible for inclusion in the analysis (Figure S2). Of these, 418 patients (15.2%) received darolutamide, 1898 (69.1%) enzalutamide, and 430 (15.7%) apalutamide. Baseline characteristics are reported in Table 1. Median age was 81 years for patients in the darolutamide and enzalutamide cohorts and 78 years in patients in the apalutamide cohort. Nearly all patients (84%) were being treated with ADT, and approximately half had received a first‐generation ARSI (e.g., bicalutamide) at baseline. In a sub‐analysis, baseline characteristics were generally similar between patients who did and did not receive ADT prior to the index date (Table S1). Median follow‐up time ranged from 18 to 25 months (Table 1). The majority of patients (92%–98%) were treated in a hospital with more than 200 beds. At least three‐quarters of patients in the darolutamide and apalutamide cohorts and more than two‐thirds of patients in the enzalutamide cohort were treated at a designated cancer hospital. The proportion of patients initiating 100% of the label dose was 78% for darolutamide, 58% for enzalutamide, and 62% for apalutamide. For all ARSIs, 3% of patients initiated at 25% of the label dose (Table 1).

**TABLE 1:** Baseline demographics and clinical characteristics.

Characteristic	Darolutamide (*n* = 418)	Enzalutamide (*n* = 1898)	Apalutamide (*n* = 430)	SMD^††^
Age, mean (SD), years	80 (7.55)	81 (7.91)	78 (8.06)	0.261
Index year, *n* (%)				
2020	24 (5.7)	466 (24.6)	124 (28.8)	0.454
2021	153 (36.6)	609 (32.1)	146 (34.0)	
2022	174 (41.6)	645 (34.0)	124 (28.8)	
2023	67 (16.0)	178 (9.4)	36 (8.4)	
Months from CRPC to index date				
Median (IQR)	0.0 (0.0–0.5)	0.0 (0.0–0.0)	0.0 (0.0–0.0)	
Mean (SD)	7.4 (25.7)	4.6 (17.9)	4.1 (15.5)	0.103
Treated with first‐generation ARSI at baseline, *n* (%)	240 (57.4)	1059 (55.8)	219 (50.9)	0.087
Treated with ADT, *n* (%)	371 (88.8)	1559 (82.1)	369 (85.8)	0.126
Initiation dose as a percentage of label dose^†^, *n* (%)				
25%	14 (3.3)	65 (3.4)	13 (3.0)	0.475
50%	75 (17.9)	571 (30.1)	79 (18.4)	
75%	5 (1.2)	160 (8.4)	72 (16.7)	
100%+^‡^	324 (77.5)	1099 (57.9)	266 (61.9)	
Missing	0	3 (0.2)	0	
Months of follow‐up^§^				
Median (IQR)	18.1 (11.1–24.8)	20.9 (12.6–31.0)	24.6 (14.9–33.4)	
Mean (SD)	18.6 (8.5)	22.2 (10.9)	24.3 (10.8)	0.382
Size of hospital, *n* (%)				
≤ 199 beds	9 (2.2)	160 (8.4)	17 (4.0)	0.235
200–499 beds	226 (54.1)	1120 (59.0)	228 (53.0)	
≥ 500 beds	183 (43.8)	618 (32.6)	185 (43.0)	
Cancer hospital, *n* (%)	338 (80.9)	1321 (69.6)	322 (74.9)	0.175
Hospital department, *n* (%)				
Urology	413 (98.8)	1780 (93.8)	421 (97.9)	0.182
Other^¶^	5 (1.2)	118 (6.2)	9 (2.1)	

Abbreviations: ADT, androgen deprivation therapy; ARSI, androgen receptor signaling inhibitor; CRPC, castration‐resistant prostate cancer; IQR, interquartile range; SD, standard deviation; SMD, standardized mean difference.

^†^ Total daily dose per label: darolutamide, 1200 mg; enzalutamide, 160 mg; apalutamide, 240 mg.

^‡^ 100%+ group includes patients who received doses higher than label dose.

^§^ Arithmetic median of follow‐up times.

^¶^ Other department includes Internal Medicine, Cardiology, etc.

^††^ SMD values less than 0.2 were considered a small effect size.

**FIGURE 1:**
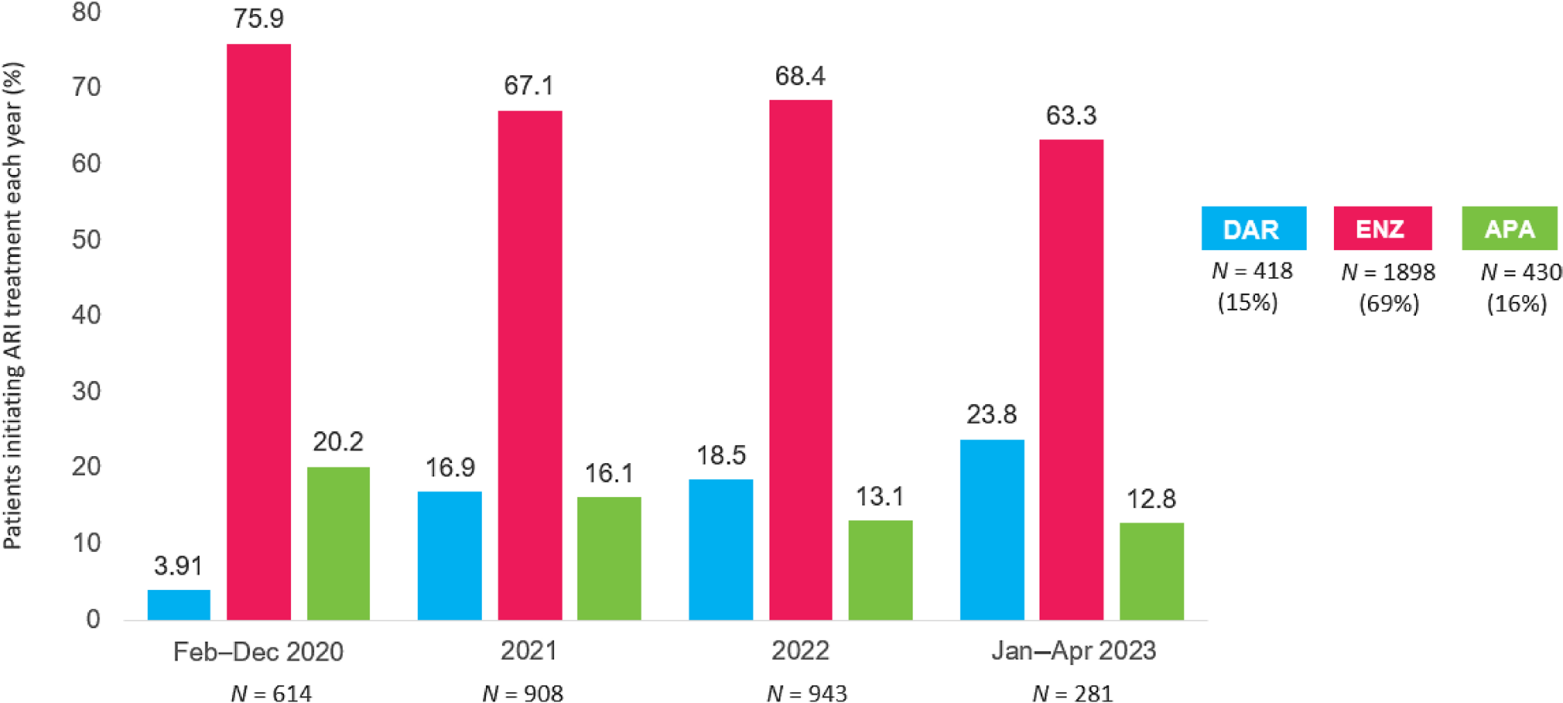
Initial ARSI starting year. APA, apalutamide; ARI, androgen receptor inhibitor; ARSI, androgen receptor signaling inhibitor; DAR, darolutamide; ENZ, enzalutamide.

**FIGURE 2:**
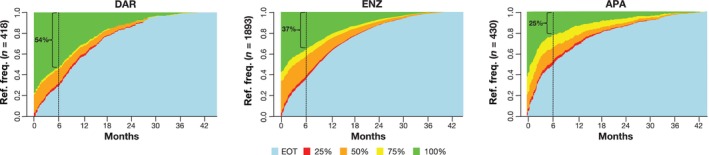
ARSI dose distribution over time. Total daily dose per label: DAR, 1200 mg; ENZ, 160 mg; APA, 240 mg. APA, apalutamide; ARSI, androgen receptor signaling inhibitor; DAR, darolutamide; ENZ, enzalutamide; EOT, end of treatment; Ref. freq., reference frequency.

In general, the proportion of patients with nmCRPC receiving darolutamide increased every year during the study period, from 3.9% to 23.8% (Figure 1). In comparison, the proportion of patients receiving enzalutamide and apalutamide decreased every year during the study period, from 75.9% to 63.3% and from 20.2% to 12.8%, respectively (Figure 1). A dose/state frequency analysis across the treatment arms showed that a greater proportion of patients receiving darolutamide initiated and stayed at the label dose, compared with those receiving enzalutamide and apalutamide (Figure 2). Note that at any time point in Figure 2, the green segment indicates the proportion treated at the label dose.

### 3.2 ARSI Discontinuation

**FIGURE 3:**
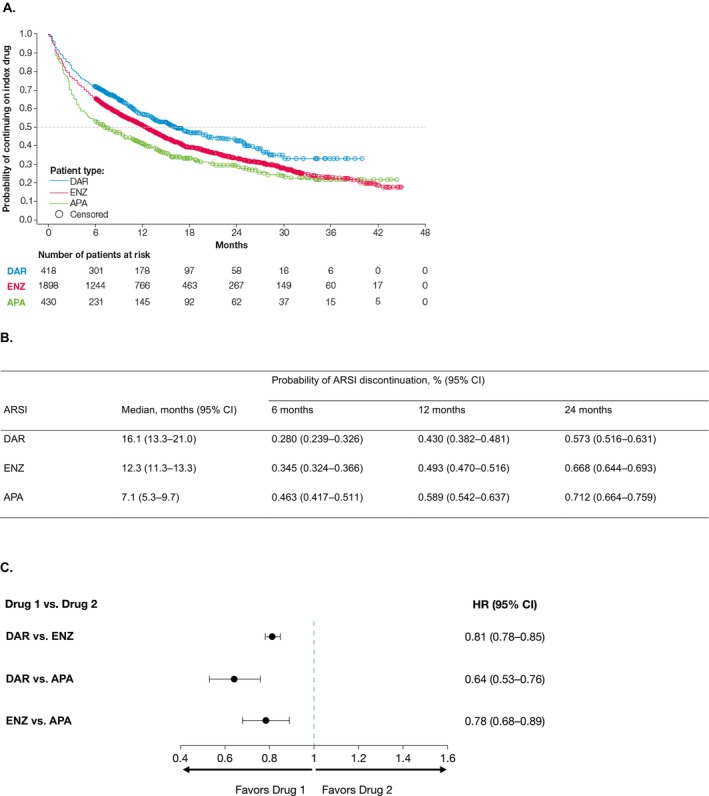
(A) Time to discontinuation of ARSI. At‐risk patient counts were calculated at the start of the time point; death was censored in this plot and is not considered as a composite endpoint. (B) Probability of ARSI discontinuation. (C) IPTW‐adjusted risk of ARSI discontinuation. APA, apalutamide; ARSI, androgen receptor signaling inhibitor; CI, confidence interval; DAR, darolutamide; ENZ, enzalutamide; HR, hazard ratio; IPTW, inverse probability of treatment weighting.

The proportion of patients who discontinued initial ARSI treatment during the study period was 51.0% (213/418), 62.6% (1188/1898), and 69.3% (298/430) for darolutamide, enzalutamide, and apalutamide, respectively. In patients who discontinued initial ARSI treatment, the most common subsequent systemic therapies other than ADT/first‐generation androgen receptor inhibitor were abiraterone acetate (16%) and docetaxel (7%) (Table S2). Median time to discontinuation was 16.1 (95% CI 13.3–21.0) months for patients receiving darolutamide, 12.3 (95% CI 11.3–13.3) months for enzalutamide, and 7.1 (95% CI 5.3–9.7) months for apalutamide (Figure 3A). KM probability estimates of treatment discontinuation were lower at each time point in the darolutamide cohort (6 months: 0.280 [95% CI 0.239–0.326]; 12 months: 0.430 [95% CI 0.382–0.481]; 24 months: 0.573 [95% CI 0.516–0.631]), followed by the enzalutamide cohort (6 months: 0.345 [95% CI 0.324–0.366]; 12 months: 0.493 [95% CI 0.470–0.516]; 24 months: 0.668 [95% CI 0.644–0.693]), then the apalutamide cohort (6 months: 0.463 [95% CI 0.417–0.511]; 12 months: 0.589 [95% CI 0.542–0.637]; 24 months: 0.712 [95% CI 0.664–0.759]) (Figure 3B). The HRs derived from weighted Cox proportional hazards models (i.e., IPTW) for time to ARSI discontinuation were 0.81 (95% CI 0.78–0.85) for darolutamide versus enzalutamide, 0.64 (95% CI 0.53–0.76) for darolutamide versus apalutamide, and 0.78 (95% CI 0.68–0.89) for enzalutamide versus apalutamide (Figure 3C). The sensitivity analysis for time to discontinuation, which excludes patients who did not receive ADT before the index date, is shown in Table S3 and aligns with the primary analysis.

### 3.3 Effectiveness Outcomes: Time to mCRPC

**FIGURE 4:**
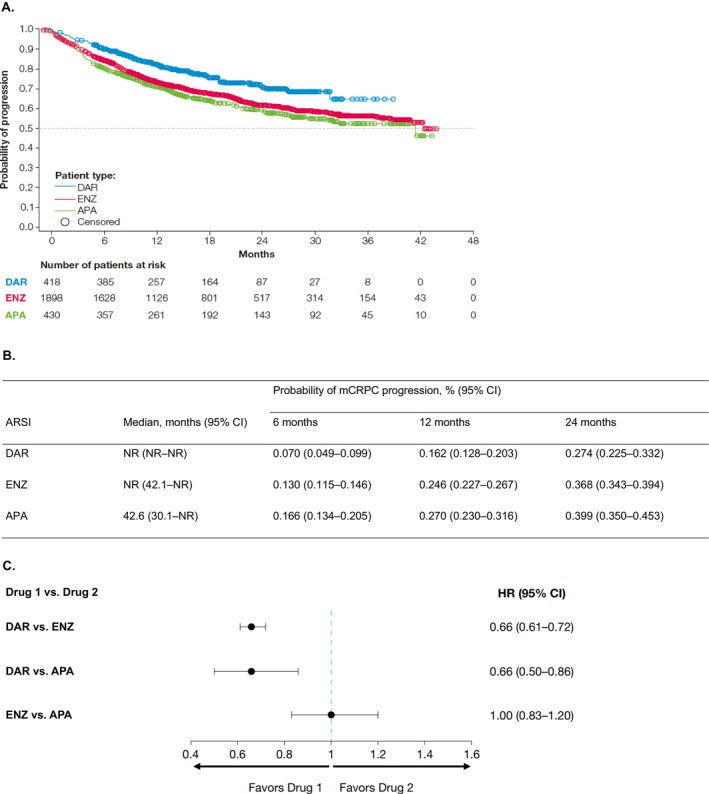
(A) Time to progression to mCRPC by ARSI. At‐risk patient counts were calculated at the start of the time point. (B) Probability of mCRPC progression. (C) IPTW‐adjusted risk of progression to mCRPC. APA, apalutamide; ARSI, androgen receptor signaling inhibitor; CI, confidence interval; DAR, darolutamide; ENZ, enzalutamide; HR, hazard ratio; IPTW, inverse probability of treatment weighting; mCRPC, metastatic castration‐resistant prostate cancer; NR, not reached.

The overall proportion of patients who progressed to mCRPC was 21.8% for darolutamide (91/418), 32.7% for enzalutamide (621/1898), and 38.1% for apalutamide (164/430) (Figure 4A). KM probability estimates of mCRPC progression were lower at each time point in the darolutamide cohort (6 months: 0.070 [95% CI 0.049–0.099]; 12 months: 0.162 [95% CI 0.128–0.203]; 24 months: 0.274 [95% CI 0.225–0.332]), compared with enzalutamide (6 months: 0.130 [95% CI 0.115–0.146]; 12 months: 0.246 [95% CI 0.227–0.267]; 24 months: 0.368 [95% CI 0.343–0.394]), and apalutamide (6 months: 0.166 [95% CI 0.134–0.205]; 12 months: 0.270 [95% CI 0.230–0.316]; 24 months: 0.399 [95% CI 0.350–0.453]) (Figure 4B). The HRs derived from weighted Cox proportional hazards models (i.e., IPTW) for time to mCRPC were 0.66 (95% CI 0.61–0.72) for darolutamide versus enzalutamide, 0.66 (95% CI 0.50–0.86) for darolutamide versus apalutamide, and 1.00 (95% CI 0.83–1.20) for enzalutamide versus apalutamide (Figure 4C). Data on the composite endpoint (time to discontinuation/progression) are provided in Supporting Information 1 and Figure S3A/B. The sensitivity analysis for time to mCRPC, excluding patients without ADT before the index date (Table S4), showed consistent findings with the primary analysis.

## 4 Discussion

ARASHI is the first large cohort study in Japan providing real‐world data on the use and outcomes of all three ARSIs indicated for the treatment of nmCRPC. The extensive MDV health claims database includes over 45 million patients, equating to 36% of the entire Japanese population, and more than 480 advanced treatment hospitals in Japan. This is highlighted by the large study sample of nearly 2750 patients who initiated ARSI treatment between February 2020 and April 2023.

In ARASHI, baseline patient characteristics were generally similar across cohorts, although the median age was lower by 3 years in the apalutamide cohort than in the other treatment groups. In addition, as a greater proportion of patients initiated apalutamide at an earlier time point between 2020 and 2022 compared with those initiating darolutamide, the follow‐up period was longer for the apalutamide cohort by 6.6 months. The ARASHI study shows that a greater proportion of patients initiated darolutamide treatment at the dose recommended in the product label compared with the other two cohorts and that a greater proportion of patients persisted on this dose during the study (Table 1; Figure 2). In addition, patients starting darolutamide had a longer median time to treatment discontinuation (16 months) compared with enzalutamide (12 months) and apalutamide (7 months). These results remained robust after adjustment for confounding factors.

Consistent with the reduced rate of discontinuations, lower proportions of patients receiving darolutamide had progressed to mCRPC by 6, 12, and 24 months compared with enzalutamide and apalutamide. After adjustment for baseline confounding factors, the risk of progression to mCRPC was still lower with darolutamide compared with enzalutamide or apalutamide. The median time to composite event of ARSI discontinuation or progression to mCRPC was also increased with darolutamide by approximately 6 months compared with enzalutamide and by 9 months compared with apalutamide.

The results seen in this Japanese population are consistent with the similar real‐world DEAR‐EXT study of 1375 patients with nmCRPC in the US, in which darolutamide was associated with a range of risk reductions of 27%–37% for ARSI treatment discontinuation and progression to mCRPC, compared with enzalutamide and apalutamide [18].

Previous real‐world studies have demonstrated the effectiveness of enzalutamide and apalutamide in treating prostate cancer in clinical practice in Japan [22, 23]. In addition, a real‐world analysis in Japanese patients with metastatic hormone‐sensitive prostate cancer revealed similar clinical outcomes with enzalutamide and apalutamide, including time to CRPC and OS, although some differences were noted between abiraterone and apalutamide [24].

The findings from ARASHI should be interpreted with caution due to limitations inherent to retrospective claims database studies, including the potential for selection bias, confounding, and other unmeasured risk factors. Some clinical baseline measures were not available within the MDV (prostate‐specific antigen values, Gleason Score, primary therapy information, and progression notes), which are important for accurately identifying the population of patients with nmCRPC and progression to mCRPC. In the absence of these clinical measures, the study relied on a validated, composite approach to define disease stage and progression, as used in other real‐world studies [25, 26]; however, misclassification may persist. Furthermore, diagnosis of CRPC was dependent on accurate and timely reporting by clinicians, although we do not expect differential reporting across ARSIs. There is also a likelihood of inaccurate and incomplete coding, or delayed documentation. Finally, data were limited to hospitals participating in the MDV.

Despite these limitations, the large study population provides generalizable insights into patients with nmCRPC across Japanese healthcare settings, with findings remaining robust across sensitivity analyses.

In conclusion, a greater proportion of patients who received darolutamide initiated treatment at label dose and appeared to continue treatment for longer than those receiving other ARSIs. Finally, progression to mCRPC appeared to be delayed in patients who received darolutamide. While these findings offer meaningful real‐world insights into ARSI use and outcomes, they should be interpreted with caution, as randomized controlled trial evidence remains essential to inform treatment decisions.

## Author Contributions

**Kazuhiro Suzuki:** supervision, conceptualization, investigation, resources, writing – review and editing. **Nasreen Khan:** supervision, conceptualization, investigation, resources, writing – review and editing. **Tomoyuki Taguchi:** supervision, conceptualization, investigation, resources, writing – review and editing. **Kana Hattori:** supervision, conceptualization, investigation, resources, writing – review and editing. **Guifang Chen:** supervision, conceptualization, investigation, resources, writing – review and editing. **Mercedeh Ghadessi:** supervision, conceptualization, investigation, resources, writing – review and editing. **Niculae Constantinovici:** supervision, conceptualization, investigation, resources, writing – review and editing. **Vanessa Quintero:** supervision, conceptualization, investigation, resources, writing – review and editing. **Hiroyoshi Suzuki:** supervision, conceptualization, investigation, resources, writing – review and editing.

## Disclosure

Approval of the Research Protocol by an Institutional Review Board: The protocol for this study was approved by suitably constituted Ethics Committees, and it conforms to the provisions of the Declaration of Helsinki and the “Ethical Guidelines for Life Science and Medical Research Involving Human Subjects” issued by the Ministry of Education, Culture, Sports, Science and Technology, the Ministry of Health, Labor and Welfare, and the Ministry of Economy, Trade and Industry in Japan.

Registry and the Registration Number of the Study: Not applicable.

Animal Studies: Not applicable.

## Consent

The authors have nothing to report.

## Conflicts of Interest

**Kazuhiro Suzuki** reports honoraria and serving as an adviser for Astellas, AstraZeneca, Bayer, Janssen, and Sanofi. **N** **asreen Khan**, **Tomoyuki Taguchi**, **Kana Hattori**, **Guifang Chen**, **Mercedeh Ghadessi**, **Niculae Constantinovici**, and **Vanessa Quintero** all report employment with Bayer. **Hiroyoshi Suzuki** reports receiving fees, honoraria, and remuneration from Bayer and Sanofi. **Kazuhiro Suzuki** and **Hiroyoshi Suzuki** are Editorial Board members of the International Journal of Urology and co‐authors of this article. To minimize bias, they were excluded from all editorial decision‐making related to the acceptance of this article for publication.

## Supporting information


Time to initial ARSI discontinuation or mCRPC progression (composite endpoint) and ‘Sensitivity analysis’.
**Figure S1:** Study design.
**Figure S2:** Patient flow.
**Figure S3:** Composite events.
**Table S1:** Baseline characteristics among patients with and without ADT before the index date.
**Table S2:** Subsequent systemic treatment among patients who discontinued index treatment.
**Table S3:** KM median time to discontinuation excluding patients without evidence of ADT before the index date.
**Table S4:** KM median time to progression to mCRPC excluding patients without evidence of ADT before the index date.
