# Supplementary material for: Androgen Receptor Signaling Inhibitors in Non‐Metastatic Castration‐Resistant Prostate Cancer in Japan: The ARASHI Study
Source: Int J Urol. 2025 Oct 3;32(12):1853–61. doi: 10.1111/iju.70235 (PMC12687924; doi:10.1111/iju.70235)
Supplement: Supplementary file 1 — Time to initial ARSI discontinuation or mCRPC progression (composite endpoint) and ‘Sensitivity analysis’. Figure S1: Study design. Figure S2: Patient flow. Figure S3: Composite events. Table S1: Baseline characteristics among patients with and without ADT before the index date. Table S2: Subsequent systemic treatment among patients who discontinued index treatment. Table S3: KM median time to discontinuation excluding patients without evidence of ADT before the index date. Table S4: KM median time to progression to mCRPC excluding patients without evidence of ADT before the index date. [file IJU-32-1853-s001.docx]

# Androgen Receptor Signaling Inhibitors in Non-Metastatic Castration-Resistant Prostate Cancer in Japan: The ARASHI Study

## SUPPORTING INFORMATION

#### Time to initial androgen receptor signaling inhibitor discontinuation or metastatic castration-resistant prostate cancer progression (composite endpoint)

## The overall proportion of patients who experienced a composite event (time to discontinuation/progression) was 53.1% for darolutamide treatment (222/418), 67.7% for enzalutamide (1285/1898), and 75.1% for apalutamide (323/430) (Figure S3A). Median time to the composite event was 15.2, 9.7, and 5.9 months for patients receiving darolutamide, enzalutamide, and apalutamide, respectively, and patients receiving darolutamide had a lower risk of experiencing a composite event over time. Kaplan–Meier probability estimates of composite event were lower at each time point in the darolutamide cohort (6 months: 0.301 [95% confidence interval [CI] 0.260–0.348]; 12 months: 0.457 [95% CI 0.408–0.508]; 24 months: 0.593 [95% CI 0.537–0.650]), compared with enzalutamide (6 months: 0.394 [95% CI 0.372–0.416]; 12 months: 0.550 [0.527–0.573]; 24 months: 0.725 [0.701–0.749]), and apalutamide (6 months: 0.502 [95% CI 0.456–0.550]; 12 months: 0.652 [95% CI 0.606–0.698]; 24 months: 0.785 [95% CI 0.739–0.828]) (Figure S3B).

#### Sensitivity analysis

An analysis was conducted in which mortality was removed from the list of events comprising the primary outcome (androgen receptor signaling inhibitor discontinuation) and treated as a censoring event. Owing to the low number of instances in which death preceded treatment discontinuation/switch (darolutamide: *n* = 7 [1.7%]; enzalutamide: *n* = 60 [3.2%]; apalutamide: *n* = 4 [0.9%]), the results did not substantially differ from those of the primary analysis. Median time to discontinuation was greatest in the darolutamide cohort (17.2 months [95% CI 13.6–24.0]), followed by enzalutamide (13.1 months [95% CI 12.2–14.2]), then apalutamide (7.2 months [95% CI 5.5–9.7]).

**FIGURE S1** Study design.


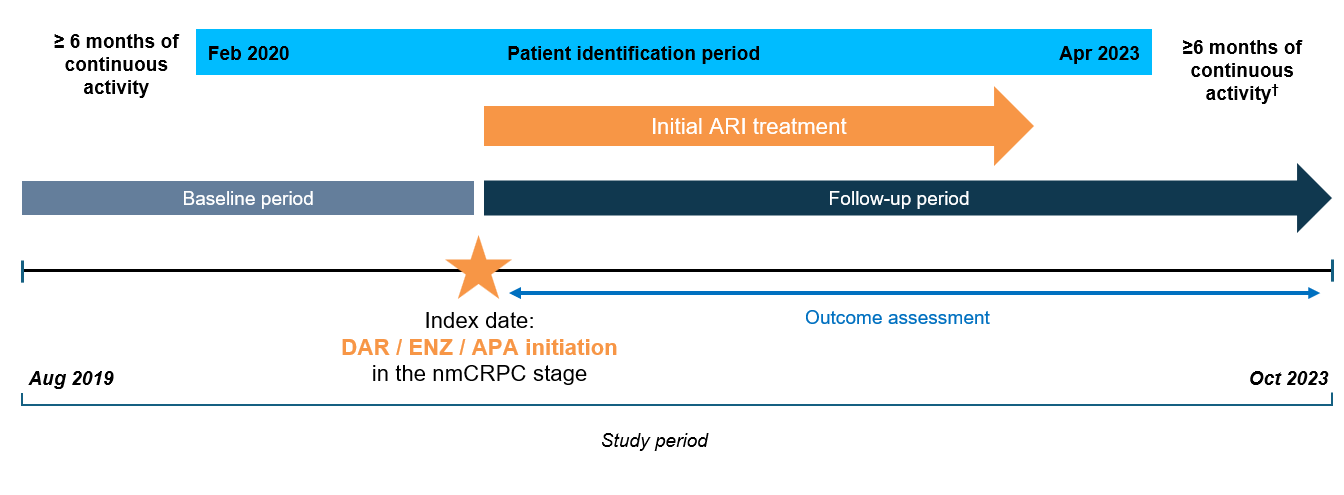
 ^†^Follow-up period: time from index date measured as continuous activity in the Medical Data Vision database and up to the end of study period, or death, whichever occurred first. In case of death, length of follow-up could be shorter than 6 months.

APA, apalutamide; ARI, androgen receptor inhibitor; ARSI, androgen receptor signaling inhibitor; DAR, darolutamide; ENZ, enzalutamide; nmCRPC, non-metastatic castration-resistant prostate cancer.

**FIGURE S2** Patient flow.


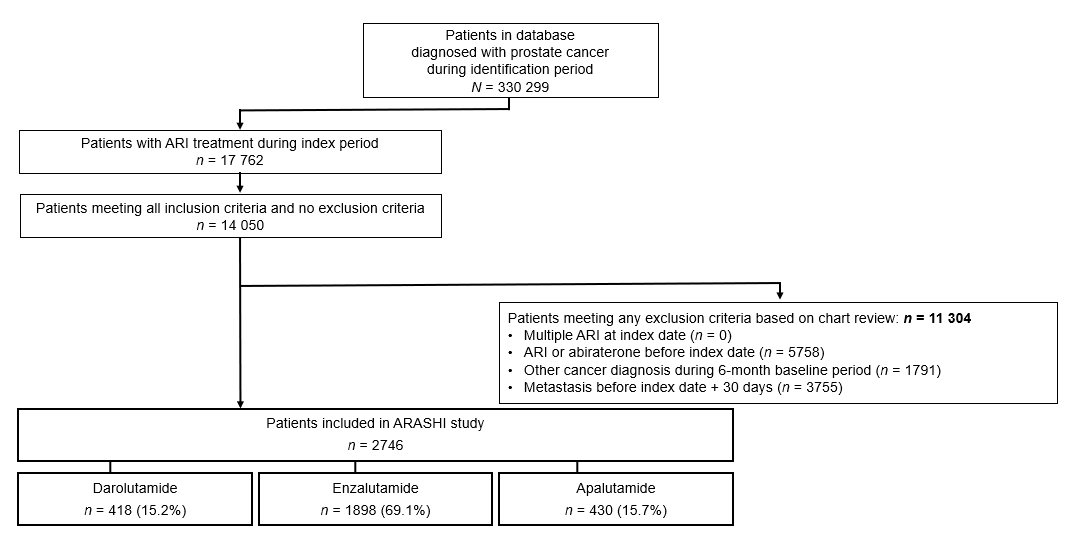


ARI, androgen receptor inhibitor; ARSI, androgen receptor signaling inhibitor.

**FIGURE S3** Composite events. (A) Time to initial ARSI discontinuation/mCRPC progression (composite^†^). At-risk patient counts were calculated as at the start of the time point. (B) Probability of composite event.

(A)


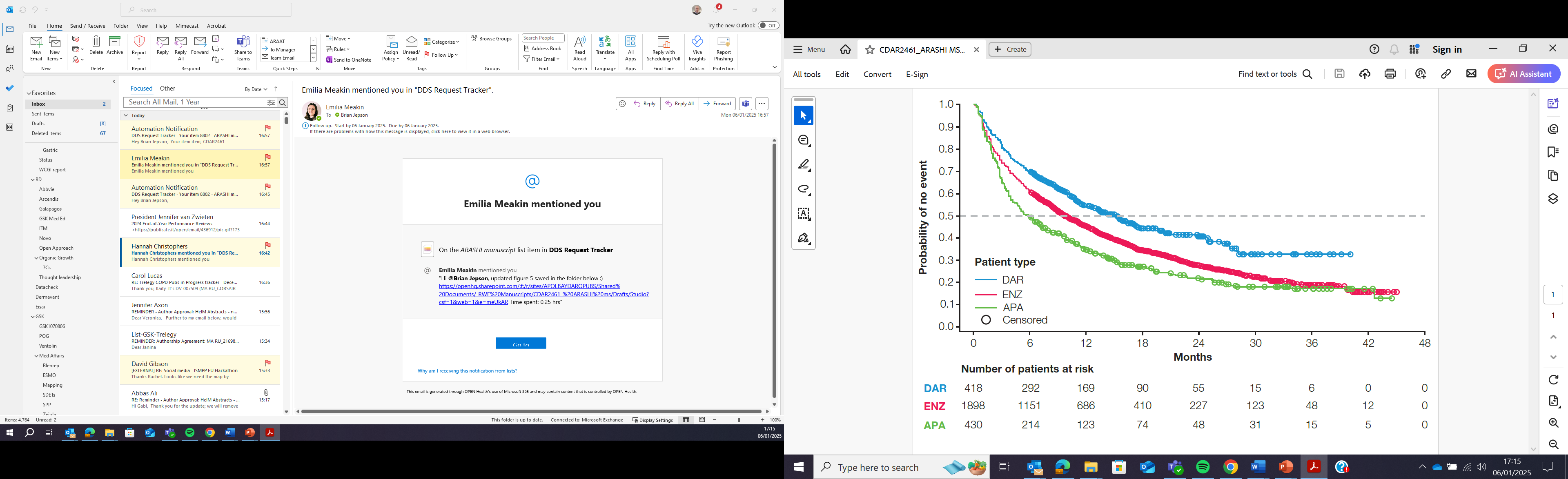


(B)

| ARSI | Median (95% CI), months | Probability of composite event, % | | |
| --- | --- | --- | --- | --- |
|  |  | 6 months | 12 months | 24 months |
| Darolutamide | 15.2 (11.4–17.6) | 0.301 (0.260–0.348) | 0.457 (0.408–0.508) | 0.593 (0.537–0.650) |
| Enzalutamide | 9.7 (8.9–10.8) | 0.394 (0.372–0.416) | 0.550 (0.527–0.573) | 0.725 (0.701–0.749) |
| Apalutamide | 5.9 (4.7–7.1) | 0.502 (0.456–0.550) | 0.652 (0.606–0.698) | 0.785 (0.739–0.828) |

^†^Composite event defined as any of the following, whichever occurred first: discontinuation, switch, progression, death.

APA, apalutamide; ARSI, androgen receptor signaling inhibitor; CI, confidence interval; DAR, darolutamide; ENZ, enzalutamide; mCRPC, metastatic castration-resistant prostate cancer.

**TABLE S1** Baseline characteristics among patients with and without ADT before the index date**.**

| **Characteristic** | **Patients with prior ADT**  **(*n* = 2299)** | **Patients without prior ADT**  **(*n* = 447)** | **SMD^††^** |
| --- | --- | --- | --- |
| **Age**, mean (SD), years | 80 (7.9) | 80 (8.2) | 0.051 |
| **Index year**, *n* (%) |  |  | 0.048 |
| 2020 | 517 (22.5) | 97 (21.7) |  |
| 2021 | 765 (33.3) | 143 (32.0) |  |
| 2022 | 781 (34.0) | 162 (36.2) |  |
| 2023 | 236 (10.3) | 45 (10.1) |  |
| **Months from CRPC to index date**  Mean (SD)  Median (IQR) | 5.4 (19.7)  0.0 (0.0–0.0) | 2.5 (14.9)  0.0 (0.0–0.2) | 0.165 |
| **Treated with first-generation ARSI at baseline,** *n* (%) | 1433 (62.3) | 85 (19.0) | 0.982 |
| **Initiation dose as a percentage of label dose**^†^, *n* (%) |  |  | 0.114 |
| 25% | 73 (3.2) | 19 (4.3) |  |
| 50% | 612 (26.6) | 113 (25.3) |  |
| 75% | 199 (8.7) | 38 (8.5) |  |
| 100%+^‡^ | 1414 (61.5) | 275 (61.5) |  |
| **Months of follow-up**^§^  Mean (SD) Median (IQR) | 22.0 (10.6)  20.9 (12.7–30.2) | 21.9 (10.9)  20.5 (12.6–31.1) | 0.010 |
| **Size of hospital**, *n* (%) |  |  | 0.085 |
| ≤ 199 beds | 152 (6.6) | 34 (7.6) |  |
| 200–499 beds | 1307 (56.9) | 267 (59.7) |  |
| ≥ 500 beds | 840 (36.5) | 146 (32.7) |  |
| **Cancer hospital**, *n* (%) | 1671 (72.7) | 310 (69.4) | 0.074 |
| **Hospital department**, *n* (%) |  |  | 0.693 |
| Urology | 2268 (98.7) | 346 (77.4) |  |
| Other^¶^ | 31 (1.3) | 101 (22.6) |  |

^†^Total daily dose per label: darolutamide, 1200 mg; enzalutamide, 160 mg; apalutamide, 240 mg.

^‡^100%+ group includes patients who received doses higher than label dose.

^§^Arithmetic median of follow-up times.

^¶^Other department includes Internal Medicine, Cardiology, etc.

^††^SMD values less than 0.2 were considered a small effect size.
ADT, androgen deprivation therapy; ARSI, androgen receptor signaling inhibitor; CRPC, castration-resistant prostate cancer; IQR, interquartile range; SD, standard deviation; SMD, standardized mean difference.

**TABLE S2** Subsequent systemic treatment among patients who discontinued index treatment**.**

| **Subsequent systemic treatment, *n* (%)** | **Darolutamide  (*n* = 213)** | **Enzalutamide  (*n* = 1188)** | **Apalutamide  (*n* = 298)** | **Total**  **(*N* = 1699)** |
| --- | --- | --- | --- | --- |
| ARSI  Abiraterone acetate  Darolutamide  Enzalutamide  Apalutamide | 40 (19)  27 (13)  0  11 (5)  2 (1) | 247 (21)  209 (18)  13 (1)  0  25 (2) | 72 (24)  44 (15)  11 (4)  17 (6)  0 | 359 (21)  280 (16)  24 (1)  28 (2)  27 (2) |
| Chemotherapy  Cabazitaxel acetonate  Docetaxel | 15 (7)  1 (1)  14 (7) | 92 (8)  2 (< 1)  90 (8) | 17 (6)  0  17 (6) | 124 (7)  3 (< 1)  121 (7) |
| Immunotherapy | 0 | 1 (< 0.1) | 0 | 1 (< 0.1) |
| PARP inhibitor | 0 | 3 (0.3) | 0 | 3 (0.2) |
| Radiotherapy | 1 (0.5) | 6 (0.5) | 0 | 7 (0.4) |
| ADT/FGARI | 86 (40) | 333 (28) | 144 (48) | 563 (33) |
| No life-prolonging prostate cancer treatment recorded in available follow-up period | 71 (33) | 506 (43) | 65 (22) | 642 (38) |

ADT, androgen deprivation therapy; ARSI, androgen receptor signalling inhibitor; FGARI, first-generation androgen receptor inhibitor; PARP, poly-ADP ribose polymerase.

**TABLE S3** KM median time to discontinuation excluding patients without evidence of ADT before the index date.

|  | **Patients with ADT before index date**  **(*n* = 2299)** | | |
| --- | --- | --- | --- |
|  | **DAR**  **(*n* = 371)** | **ENZ**  **(*n* = 1559)** | **APA**  **(*n* = 369)** |
| Median time to discontinuation, months (95% CI) | 17.0 (13.3–24.0) | 13.9 (12.9–15.2) | 8.3 (6.1–11.0) |

ADT, androgen deprivation therapy; APA, apalutamide; CI, confidence interval; DAR, darolutamide; ENZ, enzalutamide; KM, Kaplan–Meier.

**TABLE S4** KM median time to progression to mCRPC excluding patients without evidence of ADT before the index date.

|  | **Patients with ADT before index date**  **(*n* = 2299)** | | |
| --- | --- | --- | --- |
|  | **DAR**  **(*n* = 371)** | **ENZ**  **(*n* = 1559)** | **APA**  **(*n* = 369)** |
| Median time to progression to mCRPC, months (95% CI) | NR  (NR–NR) | 43.5  (42.1–NR) | 42.6  (33.2–NR) |

ADT, androgen deprivation therapy; APA, apalutamide; CI, confidence interval; DAR, darolutamide; ENZ, enzalutamide; KM, Kaplan–Meier; mCRPC, metastatic castration-resistant prostate cancer; NR, not reached.
